# PKP/PVP combine chemotherapy in the treatment of multiple myeloma patients with vertebral pathological fractures: minimum 3-year follow-up of 108 cases

**DOI:** 10.1186/s13018-019-1078-0

**Published:** 2019-02-11

**Authors:** Xingchen Yao, Ziyu Xu, Xinru Du

**Affiliations:** Department of Orthopaedics, Beijing Chao-Yang Hospital, Capital Medical University, No.8 Gongren Tiyuguan Nanlu, Chaoyang district, Beijing, China

**Keywords:** Multiple myeloma, Percutaneous kyphoplasty, Prognosis, Spine

## Abstract

**Background:**

Multiple myeloma (MM) is a blood system malignant tumor, which often leads to osteolytic bone destruction, and the vertebral column is the primary site of involvement. However, the efficacy and prognosis of percutaneous kyphoplasty/vertebroplasty (PKP/PVP) for simple vertebral pathological fractures in patients with multiple myeloma are not yet unified. The primary objective of this study was to investigate the efficacy and prognosis of PKP/PVP in the treatment of multiple myeloma patients with vertebral pathological fractures.

**Methods:**

A total of 108 patients with MM from Beijing Chao-Yang Hospital from 2007 to 2013 were enrolled. Of these, 52 patients underwent PKP/PVP surgery and chemotherapy (surgery group) and 56 received only chemotherapy (chemotherapy group). The age, gender, International Staging System (ISS), fracture location, segment, visual analog scale (VAS), Oswestry Disability Index (ODI), comprehensive treatment satisfaction, stem cell transplantation, overall survival (OS), mortality rate, and the cause of death of patients were recorded; the mean follow-up time was 42.46 months.

**Results:**

The average age of patients in surgery and chemotherapy groups was 60.8 years and 58.1 years, and the mean survival time was 41.98 months and 43.30 months, respectively. The VAS score at 1 month and last follow-up after treatment in surgery group were significantly lower than those in the chemotherapy group (*P* < 0.05); the ODI at 1 month after treatment in the surgery group was significantly lower than that in the chemotherapy group (*P* < 0.05); no significant difference was observed in the 3-year mortality rate between surgery and chemotherapy groups. The number of patients who developed activity disorder in the surgery group was significantly less than that in the chemotherapy group (*P* < 0.05). The OS of patients in ISS stage III was significantly less than that in ISS stages I and II (*P* < 0.05).

**Conclusions:**

PKP/PVP surgery can greatly relieve the pain caused by fractures, reduce the risk of being completely bedridden and pulmonary infection, and improve the quality of life of patients; however, it did not affect mortality rate and overall survival time in patients.

**Trial registration:**

As this was a retrospective study, it did not require ethical approval; all patients had signed informed consent when they received treatment, and all treatment options were voluntary.

## Background

Multiple myeloma (MM) is a blood system malignant tumor, which often leads to osteolytic bone destruction, and the vertebral column is the primary site of metastasis [1]. With the development of disease, some patients may suffer from pathological fracture or neurological dysfunction. Thus, surgery was used to stabilize the vertebral column and improve the neurological dysfunction. When MM patients have pathological vertebral fractures but no neurological symptoms and intraspinal space occupancy, minimally invasive surgery can be performed, such as percutaneous kyphoplasty (PKP) and percutaneous vertebroplasty (PVP) [2]. Some researchers found that PKP/PVP for pathological vertebral fractures in MM patients can effectively relieve symptoms in the short term without serious consequences [3]. Some scholars took a wait-and-see attitude, who held that only treatment with chemotherapy also can relieve pain in some MM patients, but surgery may lead to the risk of recurrent fractures of adjacent vertebrae [4].

Our previous studies have shown that although the bone cement leakage rate in MM patients is higher than that in osteoporotic vertebral compression fractures, no serious complications such as neurological impairment, pulmonary embolism, and short-term adjacent vertebral fractures have occurred [5]. Although some retrospective studies have initially explored the efficacy and prognosis of minimally invasive surgery for MM pathological vertebral fractures in recent years, these studies have to some extent been inadequate, such as fewer cases included and shorter follow-up time. Thus, the benefits of minimally invasive surgery for MM patients remain to be further studied.

In this retrospective study, a 3-year follow-up study was conducted to review the patients with MM treated at the Beijing Chao-Yang Hospital, Beijing, China. We analyzed the visual analog scale (VAS), Oswestry Disability Index (ODI), postoperative activities, comprehensive treatment satisfaction, and other clinical indicators to explore the efficacy and prognosis of minimally invasive surgery for multiple myeloma complicated with pathological vertebral fractures.

## Methods

### General information

**Fig. 1:**
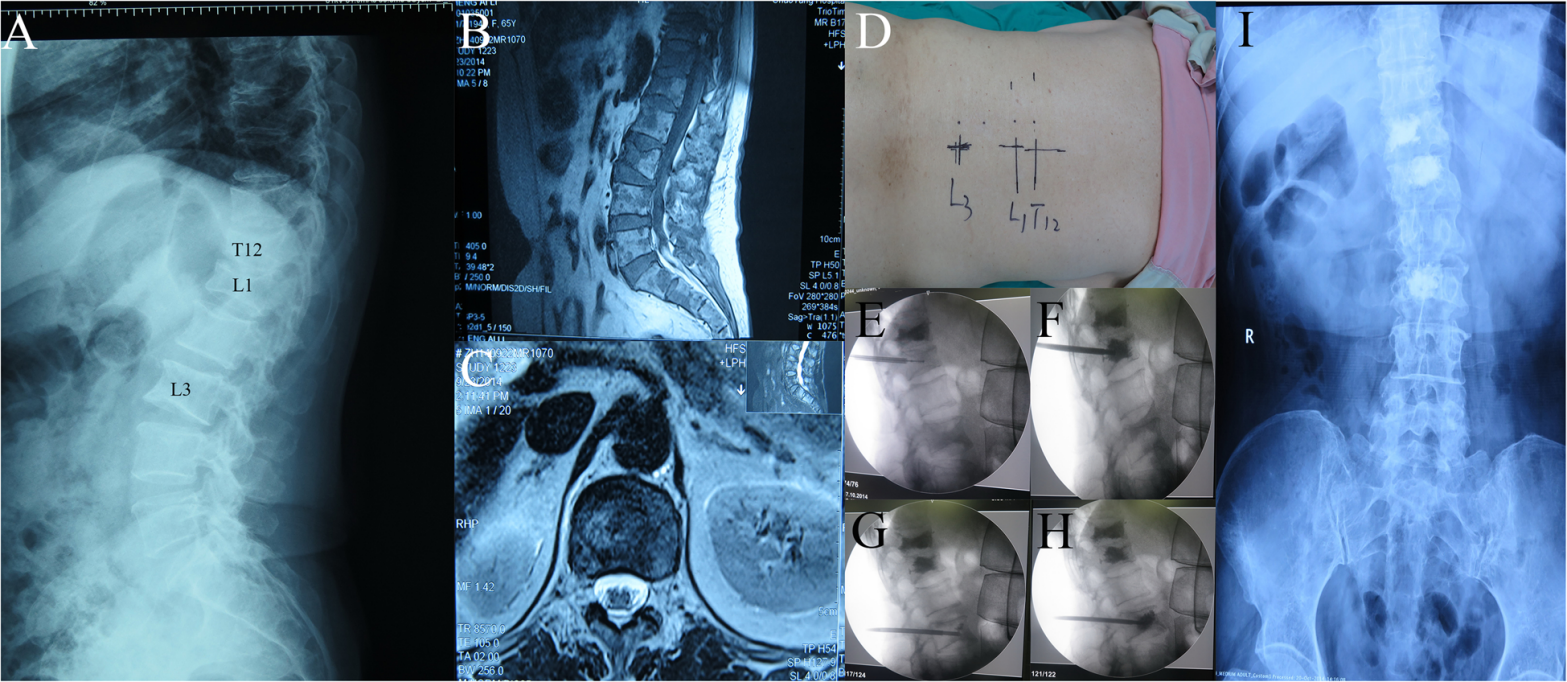
Images from one patient from the surgery group. **a**–**c** Patient with multiple myeloma with a pathological fracture of T12/L1/L3. **d**–**h** Patient was placed in the prone position, and the location of the lesion was determined by X-ray using a C-arm and marked. The filling of the vertebral body with bone cement was monitored. Image obtained during surgery. **i** A postoperative X-ray plain film scan indicated that the bone cement was implanted successfully. Image obtained following surgery

A total of 108 patients with MM treated at our hospital from 2007 to 2013 were enrolled. The pathological fracture of the vertebral column was diagnosed based on the X-ray, CT, and MRI, and the location and number of fractures were determined. All patients were > 18 years of age and had lumbar back pain without neurological symptoms. A total of 52 patients underwent PKP/PVP and chemotherapy (surgery group) (Fig. 1), and 56 patients received chemotherapy alone (chemotherapy group). The surgery group involved a total of 86 vertebrae, of which 37 were thoracic vertebrae and 49 were lumbar vertebrae. The chemotherapy group involved a total of 112 vertebrae, of which 2 were cervical vertebrae, 59 were thoracic vertebrae, and 51 were lumbar vertebrae. The age, gender, International Staging System (ISS), fracture location, segment, VAS, ODI, comprehensive treatment satisfaction, stem cell transplantation, overall survival (OS), mortality rate (1, 2, and 3 years), and the cause of death of patients were analyzed statistically. ISS stage I was defined as serum β2-microglobulin levels ≤ 3.5 mg/L and serum albumin levels ≥3.5 g/dL. ISS stage II included all patients with neither stage I nor stage III disease. ISS stage III was defined as serum β2-microglobulin levels ≥ 5.5 mg/L, irrespective of serum albumin levels [6]. Among these parameters, ISS staging was often used to assess the tumor load and prognosis of patients, which was regarded as a major risk factor affecting the survival time in patients with MM [7, 8]. Therefore, in this study, ISS staging was selected as the staging standard for statistical analysis.

Herein, the VAS, ODI, and incidence rate of pain-related activity disorders were observed before and after treatment in order to assess the quality of life. Comprehensive treatment satisfaction was used to evaluate patients’ satisfaction with treatment effects. Moreover, the prognosis of patients was assessed by the OS, 3-year mortality rate, and the cause of death of patients in each group. After the patients were divided into groups according to the ISS staging, the quantity and location of fractures, as well as the efficacy of PKP/PVP in patients with MM, were evaluated by various methods.

### Statistical methods

Patients’ clinical symptoms and data before and after the treatment were analyzed by chi-square test and independent sample *t*-test. The risk factors that might affect the OS in patients were analyzed by Cox model analysis (univariate analysis and multivariate analysis). The survival time was assessed by the Kaplan–Meier method and examined by the log-rank test. All data were analyzed by SPSS 23.0 statistical software. The measurement data were expressed as mean ± standard deviation, and *P* < 0.05 was considered as significant difference.

## Results

**Fig. 2:**
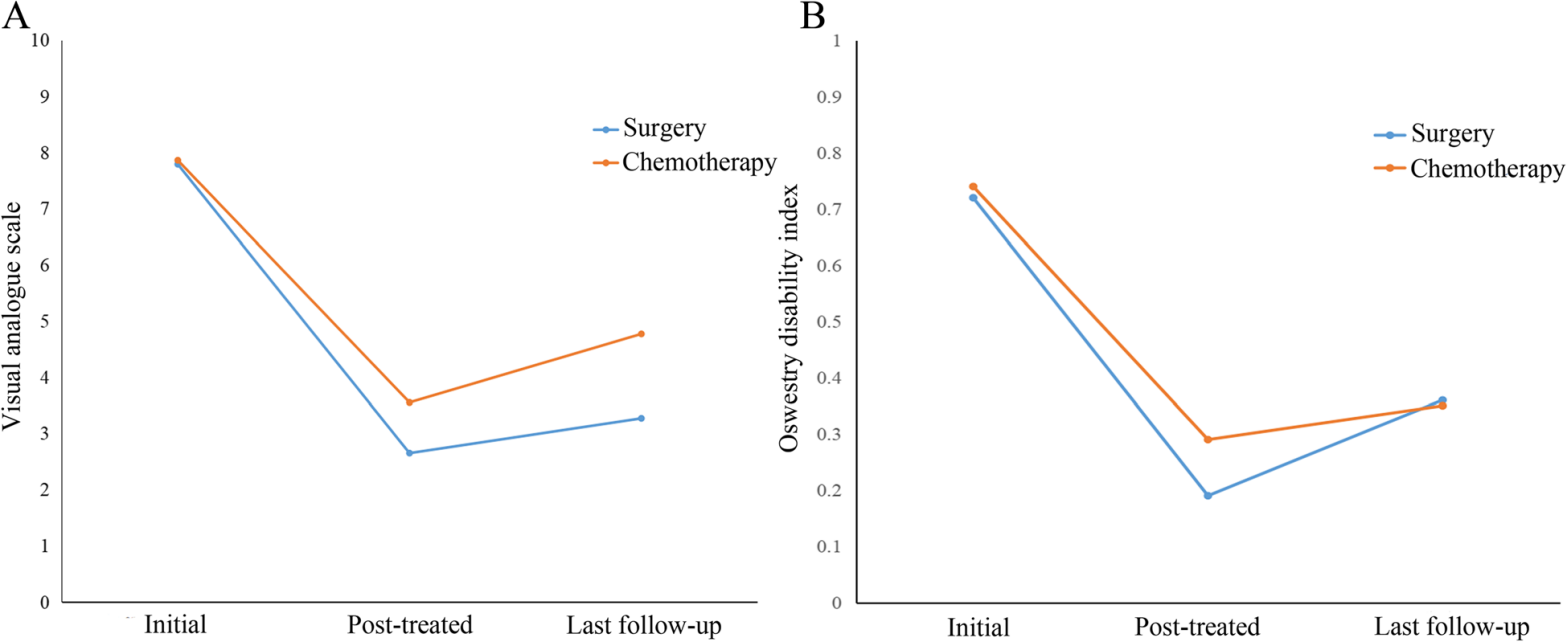
Clinical results. **a** Pain reduction at 1 month postoperatively is superior to that of the chemotherapy treatment. **b** At 1 month, the scores of ODI show a significant difference between the two groups

A total of 108 patients in the two groups were analyzed by a single factor method (Table 1). Surgery group comprised of 23 males (44.2%) and 29 females (55.8%), with an average age of 61.81 years (SD: 8.841), and 9 patients received stem cell transplantation (17.3%). The chemotherapy group consisted of 31 males (55.4%) and 25 females (44.6%), the average age was 58.14 years (SD 8.225), and 18 patients underwent stem cell transplants (32.1%). Furthermore, no statistical difference was observed in ISS staging, and the quantity and location of fractures between the two groups; the mean follow-up time was 42.5 months. The VAS score at 1 month and last follow-up after treatment in the surgery group were significantly lower than those in the chemotherapy group (2.65 ± 0.683 and 3.27 ± 0.744 vs. 3.55 ± 0.893 and 4.77 ± 1.279) (*P* < 0.001) (Fig. 2). The ODI at 1 month after treatment in the surgery group was significantly lower than that in the chemotherapy group (0.19 ± 0.072 vs. 0.29 ± 0.116) (*P* < 0.05). However, there was no significant difference in the ODI between the two groups at the last follow-up (0.36 ± 0.094 vs. 0.35 ± 0.101) (*P* > 0.05). The mean change VAS score at 1 month and last follow-up in the surgery group were significantly lower than those in the chemotherapy group (5.13 ± 0.971 and 4.52 ± 0.960 vs. 4.30 ± 0.952 and 3.09 ± 1.180) (*P* < 0.001). The mean change ODI at 1 month in the surgery group and chemotherapy group were 0.53 ± 0.156 and 0.45 ± 0.146 (*P* < 0.05), and mean change ODI at the last follow-up in the two groups were 0.36 ± 0.143 and 0.39 ± 0.149 (*P* > 0.05) (Table 1).

**Table 1:** Basic information of 108 MM patients with vertebral pathological fracture (single factor analysis)

Characteristic	Surgery value *N* (%) or [SD]	Chemotherapy value *N* (%) or [SD]	*P* value
Age	60.81 [8.841]	58.14 [8.225]	0.108
Gender			0.248
Male	23 (44.2%)	31 (55.4%)	
Female	2955.8%)	25 (44.6%)	
ISS staging			0.529
ISS-I	8 (15.4%)	9 (16.1%)	
ISS-II	22 (42.3%)	18 (32.1%)	
ISS-III	22 (42.3%)	29 (51.8%)	
Fracture quantity			0.376
1	30 (57.6%)	25 (44.6%)	
2	11 (21.2%)	17 (30.4%)	
≥ 3	11 (21.2%)	14 (25.0%)	
Fracture location			0.327
Cervical vertebra	0 (0.0%)	0 (0.0%)	
Thoracic vertebra	18 (34.6%)	24 (42.9%)	
Lumbar vertebra	24 (46.2%)	18 (32.1%)	
Short segment	10 (19.2%)	14 (25.0%)	
Stem cell transplantation	9 (17.3%)	18 (32.1%)	0.075
VAS initial	7.79 [0.637]	7.86 [0.903]	0.651
VAS post-treated			
1 month	2.65 [0.683]	3.55 [0.893]	0.000
Mean change at 1 month	5.13 [0.971]	4.30 [0.952]	0.00
Last follow-up	3.27 [0.744]	4.77 [1.279]	0.000
Mean change at last follow-up	4.52 [0.960]	3.09 [1.180]	0.000
ODI initial	0.72 [0.127]	0.74 [0.149]	0.381
ODI post-treated			
1 month	0.19 [0.072]	0.29 [0.116]	0.000
Mean change at 1 month	0.53 [0.156]	0.45 [0.146]	0.009
Last follow-up	0.36 [0.094]	0.35 [0.101]	0.719
Mean change at last follow-up	0.36 [0.143]	0.39 [0.149]	0.284
Activity disorder	4 (7.7%)	13 (23.2%)	0.027
Overall survival (month)	41.98 [22.168]	42.91 [21.312]	0.825
Mortality rate			
1 year	4 (7.7%)	1 (1.8%)	0.144
2 years	10 (19.2%)	12 (21.4%)	0.777
3 years	22 (42.3%)	23 (41.1%)	0.896
Cause of death	3 people died of pneumonia (13.6%), 18 people died of disease progression (81.8%), 1 person died of hemorrhage of the digestive tract (4.5%)	10 people died of pneumonia (43.5%), 13 people died of disease progression (56.5%)	0.027

The rates of 3-year mortality in the two groups were 7.7%, 19.2%, and 42.3% and 1.8%, 21.4%, and 41.1%, respectively (*P* > 0.05). Moreover, the analysis of the cause of death in patients in both groups revealed that the number of patients who developed a pulmonary infection in the chemotherapy group (*n* = 10) was significantly more than that in the surgery group (*n* = 3) (*P* < 0.05). The number of patients who developed an activity disorder in the surgery group (4 patients) was significantly less than that in the chemotherapy group (13 patients) (*P* < 0.05); however, no significant difference was observed in the OS between the two groups (surgery group 41.98 ± 22.168, chemotherapy group 42.91 ± 21.312) (*P* > 0.05).

The cohort of 108 patients was divided into groups according to age, ISS staging, fracture quantity, and fracture segments. The OS of patients who underwent surgery and chemotherapy was analyzed by univariate analysis, and no significant difference was observed (*P* > 0.05) (Table 2).

**Table 2:** Comparison of OS in MM patients who received surgery and Chemotherapy in different groups (Univariate analysis)

Characteristic	Overall survival (months) [SD]		*P*-value
	Surgery	Chemotherapy	
Age			
<60-year-old	40.25 [23.055]	38.61 [18.858]	0.809
>60-year-old	44.17 [25.482]	38.88 [22.193]	0.526
ISS staging			
I	50.75 [22.257]	65.56 [19.014]	0.160
II	53.55 [20.907]	49.39 [16.776]	0.499
III	27.23 [13.921]	32.62 [16.753]	0.227
Fracture quantity			
1	37.94 [23.231]	40.80 [21.348]	0.721
≥2	46.71 [24.816]	37.42 [19.478]	0.187
Fracture segment			
Single segment	42.41 [25.356]	37.36 [19.827]	0.413
Short-segment	42.00 [20.100]	42.18 [21.009]	0.986

**Fig. 3:**
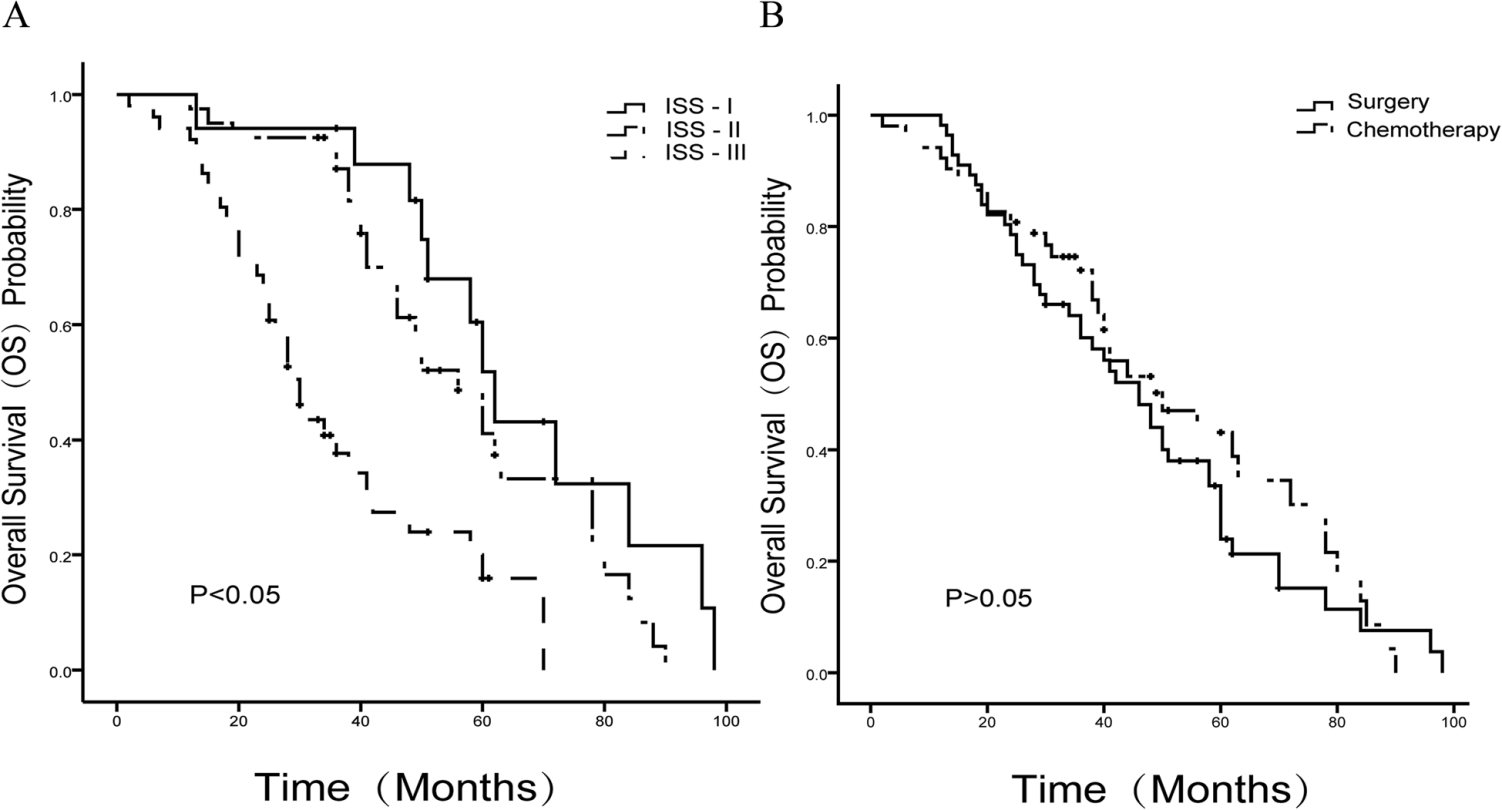
**a** Comparison of OS in patients within ISS stages I, II, and III, among which, the OS in patients within ISS stages I and II was significantly higher than that within ISS stage III (*P* < 0.001), and there was no significant difference in the OS in patients within ISS stages I and II (*P* > 0.05). **b** No significant difference in the OS in patients in the surgical and non-surgical groups (*P* > 0.05)

Multivariate analysis of all data revealed a high correlation between ISS staging and OS, and ISS staging was the only independent risk factor of OS. The OS of patients in the ISS stage III was significantly lower than that in ISS stages I and II (*P* < 0.001) (Table 3, Fig. 3).

**Table 3:** Multivariate Cox regression analysis of risk factors affecting the survival time of MM patients

Characteristic	*P*-value	HR	95% CI
Age	0.156	1.020	0.992–1.049
Gender	0.142	0.669	0.391–1.145
ISS staging			
I	0.000	0.164	0.068–0.397
II	0.000	0.314	0.166–0.593
III	1	–	–
Fracture location			
Thoracic vertebra	0.515	1.280	0.609–2.690
Lumbar vertebra	0.661	1.194	0.541–2.634
Short-segment	1	–	–
Fracture quantity			
1	0.981	1.008	0.505–2.014
2	0.900	1.043	0.539–2.020
≥3	1	–	–
Stem cell transplantation	0.771	0.914	0.499–1.674
Activity disorder	0.505	0.797	0.410–1.553
Surgery	0.595	0.863	0.500–1.488

**Fig. 4:**
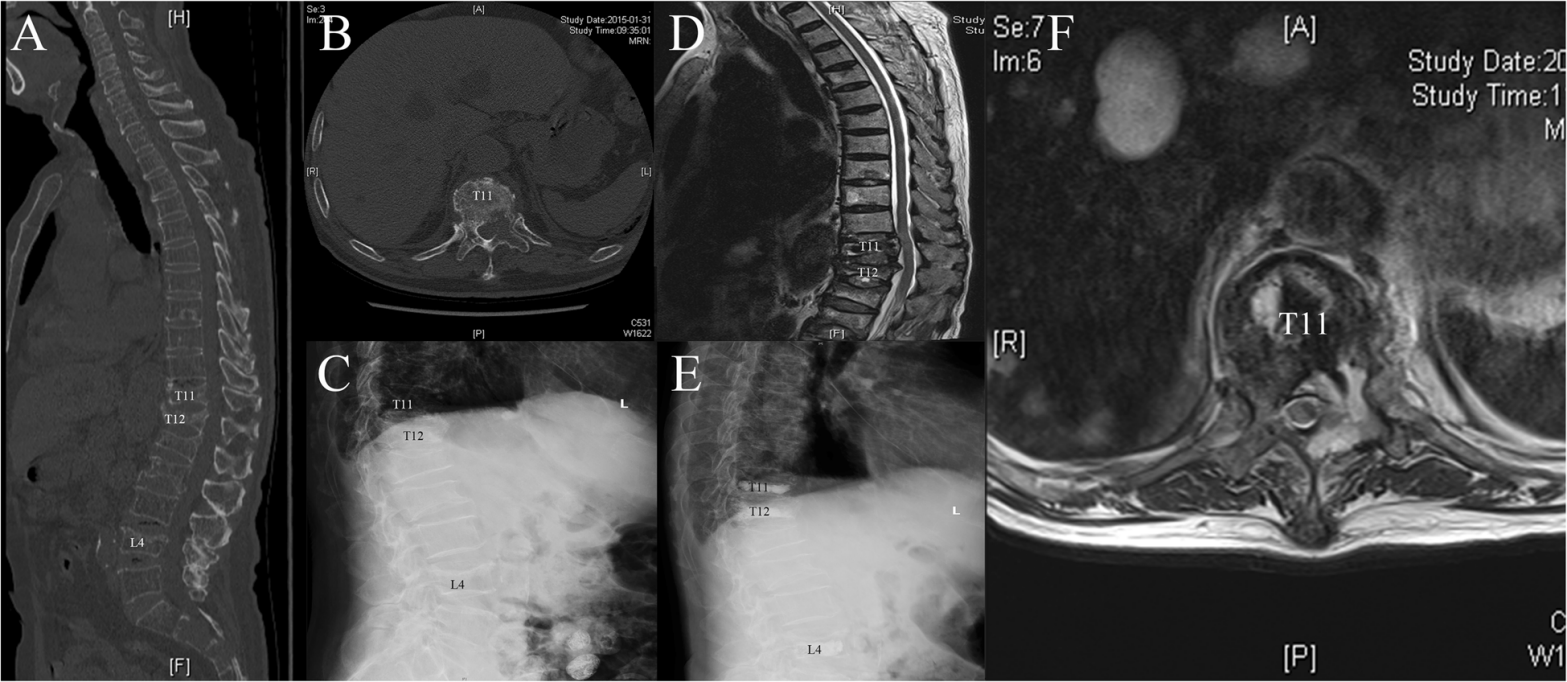
Images from one patient who developed lower extremity paralysis after surgery. **a**–**c** Patient with multiple myeloma with a pathological fracture of T12/T11/L4. CT scan showed that T11/12/L4 vertebral body osteolytic destruction, no spinal canal compression. **e** Postoperative X-ray plain film scan indicated that the bone cement were implanted successfully. **d**, **f** MRI revealed soft tissue formation in T11 vertebral body and left pedicle, and paraspinal and costal thoracic joints

In addition, all patients in the surgery group underwent surgery successfully; five patients developed complications such as bone cement leakage. The intraspinal leakage of the filling materials occurred in three patients, and paravertebral leakage of filling materials occurred in two patients; however, none of the patients displayed any obvious neurological symptoms. One patient developed lower extremity paralysis after surgery, and MRI revealed that the spinal canal was compressed by a local tumor (Fig. 4). In terms of comprehensive treatment satisfaction, patients who respond “much better” in the surgery group were higher than those in the chemotherapy group (65.4% vs. 17.9%) (*P* < 0.05) (Table 4).

**Table 4:** Analysis of comprehensive satisfaction of 108 patients after treatment (Chi-square test)

Response	Surgery Value *N* (%)	Chemotherapy Value *N* (%)	*P*-value
Very much better	34 (65.4%)	10 (17.9%)	0.000
Much better	10 (19.2%)	31 (55.4%)	0.000
A little better	5 (9.6%)	14 (25.0%)	0.065
No change	2 (3.8%)	1 (1.8%)	–
Worse	1 (1.9%)	0 (0.0)	–

## Discussion

MM is often associated with multiple vertebral fractures, and with the progression of the disease, the pathological fracture could occur easily. The role of surgery in the management of myeloma-related spinal involvement is controversial. The intraspinal-occupying lesions occurred in patients might develop spinal cord compression symptoms or paraplegia. Presently, the multidisciplinary treatment is accepted as the primary treatment for MM [9, 10]. However, for the surgical treatment, there are several key issues, including the efficacy of the treatment requires consideration before selecting the approach. Some studies [11–13] suggested that when patients presented a vertebral fracture accompanied by neurological symptoms, surgical treatment was effective in relieving pain, maintaining vertebral volume stabilization, improving the quality of life, and reducing the rate of mortality. Moreover, when a tumor constricts the vertebral canal, an immediate surgical removal is essential to relieve the compression of the vertebral canal. Previous studies [14–16] speculated that chemotherapy or radiotherapy alone could achieve the corresponding treatment effect. Strikingly, Cai [17] reviewed 30 MM patients with spinal metastasis undergone internal fixation, and the pain and neurological function of the patients were found to be improved post-surgery. The study postulated that internal fixation was efficient in the treatment of MM patients with spinal instability and neurological dysfunction. Zhang [18] analyzed 36 MM patients with vertebral canal invasion in a retrospective study and found that surgery improved the neurological symptoms of such patients. Furthermore, the early diagnosis of vertebral canal invasion seemed to be critical for patients with MM. Audat [19] who reviewed 14 MM patients who underwent vertebroplasty/kyphoplasty found that adding vertebral augmentation to conventional therapy improves multiple myeloma patients’ quality of life, but did not affect the mortality rate.

In the case of small trauma, easy operation and high safety led to an increased usage of the minimally invasive surgery in the treatment of osteolytic tumor of the spine with an excellent curative effect [4, 20]. Ha [21] followed up 27 patients with MM, who had undergone bone cement treatment and demonstrated rapid pain relief post-surgery. Julka [22] performed PKP surgery on 32 MM patients with a compression fracture of vertebral body but without nervous dysfunction. The study found that the pain was significantly improved and vertebral height was essentially restored after surgery, thereby designating it as a safe and effective method for the treatment of MM with a vertebral compression fracture. Kasperk [23] reviewed 73 patients with MM who were subjected to PKP surgery, radiotherapy, and systemic therapy alone; among these patients, the pain in the surgery group was relieved 2 years post-surgery as compared to the other two groups. Moreover, the pain-related activity disorders and the risk of refracture were significantly decreased. Mendoza [24] analyzed the cancer-related symptoms of 79 patients with MM, who underwent PKP/PVP, and found that when the patient’s pain was relieved, the anxiety, depression, and cancer-related symptoms were mitigated. The study also speculated that surgery was valuable in improving the overall well-being of the patients harboring the tumor.

For surgical treatment in MM, there were two main concerns: whether surgical treatment could improve the quality of life in patients and whether it could impact the mortality rate of patients with MM. Therefore, single factor analysis was performed to assess 108 MM patients with vertebral pathological fracture; the pain of patients in the surgery group was found to be relieved (*P* < 0.001). The number of patients who developed activity disorder in the surgical group was significantly less than that in the non-surgical group (*P* < 0.05), and the incidence of surgical complications was low. However, PKP/PVP did not prolong the OS in patients with MM according to the statistical results. In addition, the analysis of the cause of death in the two groups showed that the probability of pulmonary infection in the non-surgical group was significantly higher than that in the surgical group (*P* = 0.027). Furthermore, we proposed that pain relief in the non-surgical group was not distinct, which in turn, affected the daily activities and functional exercises in patients and increased the risk of pulmonary infection. Pneumonia and deep venous thrombosis of the lower limb were the leading causes of death in elderly individuals. When patients with MM suffered from pneumonia or poor nutritional status, they could not receive any chemotherapy; however, after patients underwent surgery, the incidence of pneumonia could be reduced, which also laid the foundation for an uninterrupted follow-up treatment. Nevertheless, the indirect effect of surgery cannot be ignored as it is crucial for the multidisciplinary treatment of MM.

Moreover, after all factors related to survival time were analyzed by multivariate analysis, age, gender, surgery, stem cell transplantation, fracture segment, fracture quantity, and the ability of activity, except ISS staging, were not found to be independent risk factors for OS in patients with MM. The OS of patients in ISS stage III was significantly less than that in ISS stages I and II (*P* < 0.001), which was in agreement with the results of the study by Amelot [7]. Moreover, a total of 108 patients were divided into groups according to their age, ISS staging, fracture quantity, and fracture segment, and single factor analysis was performed to evaluate the OS of patients with and without surgery in different groups; however, no significant difference was found in the OS of patients with and without surgery (*P* > 0.05).

According to the above results, PKP/PVP did not affect the rate of mortality and OS of MM patients in different ISS stages; the location and quantity of fractures neither increased the risk of surgery nor affected the OS in patients. However, after the patients underwent surgery, rapid relief of pain was experienced, and routine life activities were improved. Moreover, the incidence rate of pneumonia was reduced due to prolonged bed rest, following which, the MM patients recovered rapidly and could undergo the next stage of chemotherapy or radiotherapy for the overall treatment.

Nonetheless, this study presented some limitations. Firstly, chemotherapy and radiotherapy programs were not considered as the risk factors for MM survival time. Since the patients diagnosed with MM from 2007 to 2013 were enrolled in this study, the time span of the survey was prolonged. Furthermore, with continual development of the chemotherapeutic program of MM, that for different patients might be varied. Secondly, unlike other spinal malignant tumors, MM was a hematological malignancy. Patients might have also suffered from the pathological fractures of other sites and secondary organ damage to vital organs, such as the heart, liver, and kidney that might affect the prognosis of patients with MM. The purpose of this study was to evaluate the efficacy and prognosis of minimally invasive surgery for MM, and only a few cases of the secondary organ damage were noted; therefore, the impact on the survival time was not analyzed.

## Conclusion

Based on the above results, we concluded that PKP/PVP in MM patients with vertebral pathological fracture was effective in relieving pain. In addition, it reduced the incidence of complications related to bed rest, which in turn, significantly improved the quality of life in patients. Moreover, the rate of mortality and overall survival time in patients was not affected.
